# Successful Outcome Treating Pyoderma Gangrenosum and Pouchitis With Upadacitinib

**DOI:** 10.14309/crj.0000000000001442

**Published:** 2024-08-10

**Authors:** Paula Milena Prieto Jimenez, Siamak Tabib, Brook Abbott, Gil Melmed

**Affiliations:** Cedars Sinai IBD Medical Center, New York, NY

## INTRODUCTION

There are no approved therapies for pyoderma gangrenosum (PG), an uncommon but debilitating skin manifestation of inflammatory bowel diseases (IBD; ulcerative colitis [UC] and Crohn's disease [CD]). The selective Janus kinase (JAK) inhibitor, upadacitinib, was recently approved for the treatment of moderate-to-severe UC and CD. Its effectiveness in treating PG in the IBD population is unknown, although a case series describes use of the nonselective JAK inhibitor tofacitinib effectively used to treat PG in 3 patients with Crohn's disease.^1^ We describe a 40-year-old woman with a history of ulcerative colitis and subsequent colectomy with ileal-pouch anastomosis who developed J-pouch inflammation together with pyoderma gangrenosum, both of which improved with upadacitinib.

## CASE REPORT

**Figure 1.**
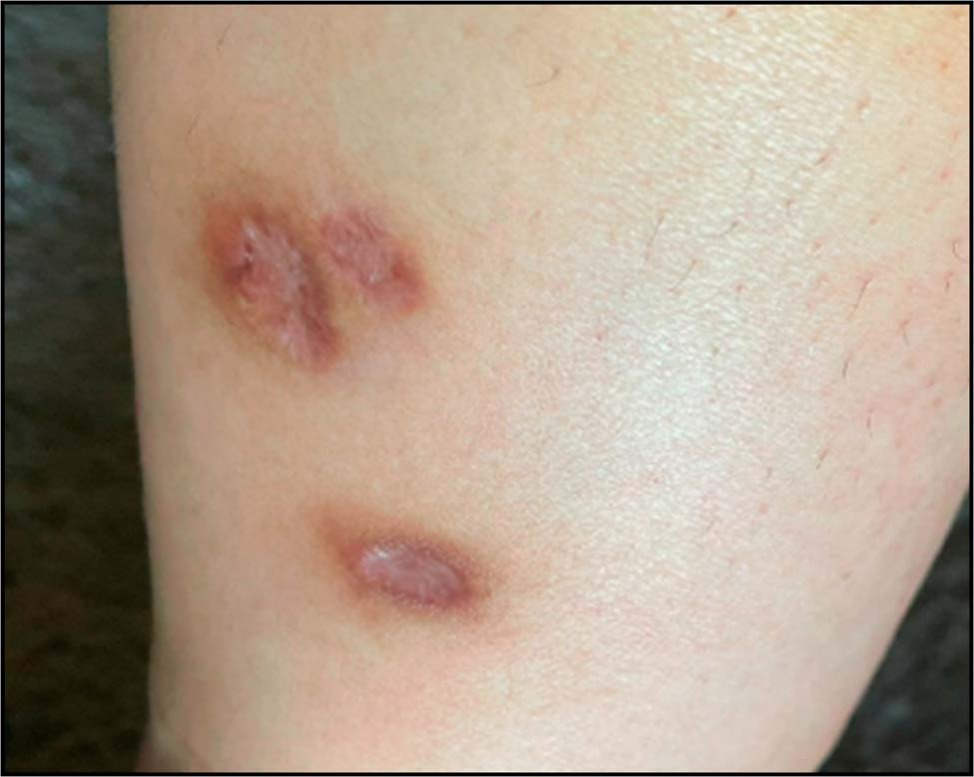
Lateral view of the left lower extremity showing classic ulcerative pyoderma gangrenosum.

**Figure 2.**
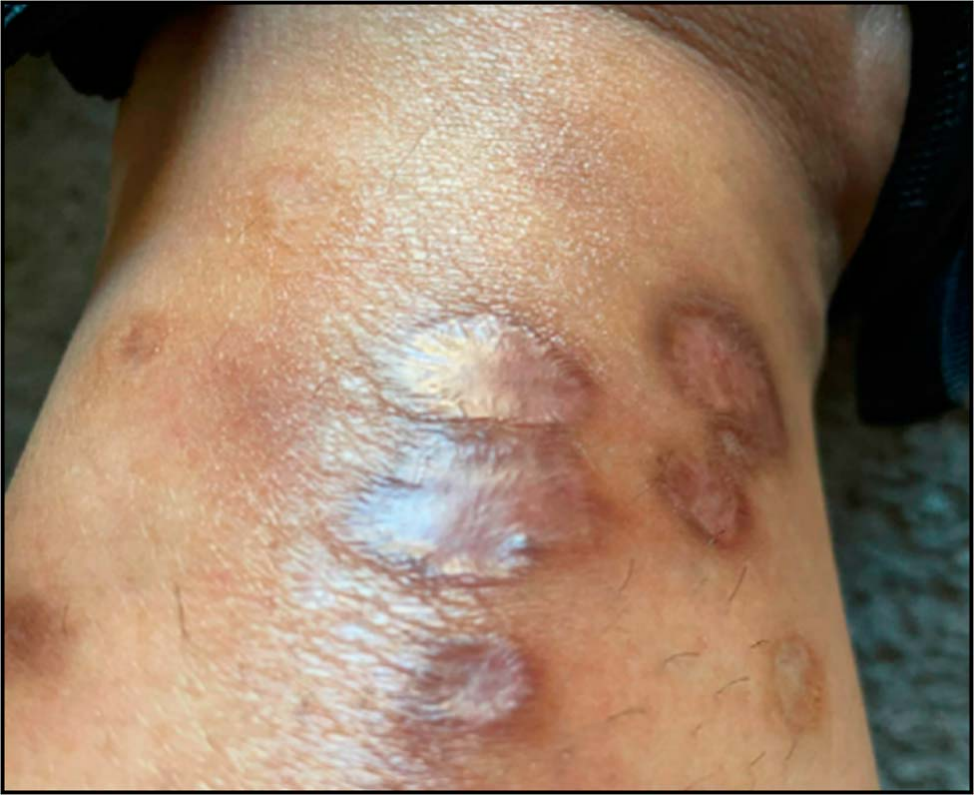
Medial view of the left lower extremity showing classic ulcerative pyoderma gangrenosum.

A 40-year-old woman presented to the emergency department with fever and multiple bilateral 1 cm ulcerated skin lesions with surrounding erythema on her bilateral lower extremities (Figures 1 and 2). She also was experiencing 4 to 5 non-bloody loose bowel movements per day, associated with intermittent nocturnal episodes, and weight loss with mild abdominal pain. She had a history of asthma, insulin resistance, and ulcerative colitis, for which she had undergone total colectomy with an ileal-pouch anastomosis for medically refractory disease many years previously.

Her inflammatory bowel disease history was notable for diagnosis at the age of 25 years. She had secondary nonresponse to anti-tumor necrosis factor infliximab in 2013, developed pouchitis in 2022, and had secondary nonresponse to ustekinumab in 2023. She had multiple extraintestinal manifestations (EIMs) including erythema nodosum, psoriasis, and sacroiliitis, all treated with dapsone 50 mg daily and leflunomide 10 mg daily.

**Figure 3.**
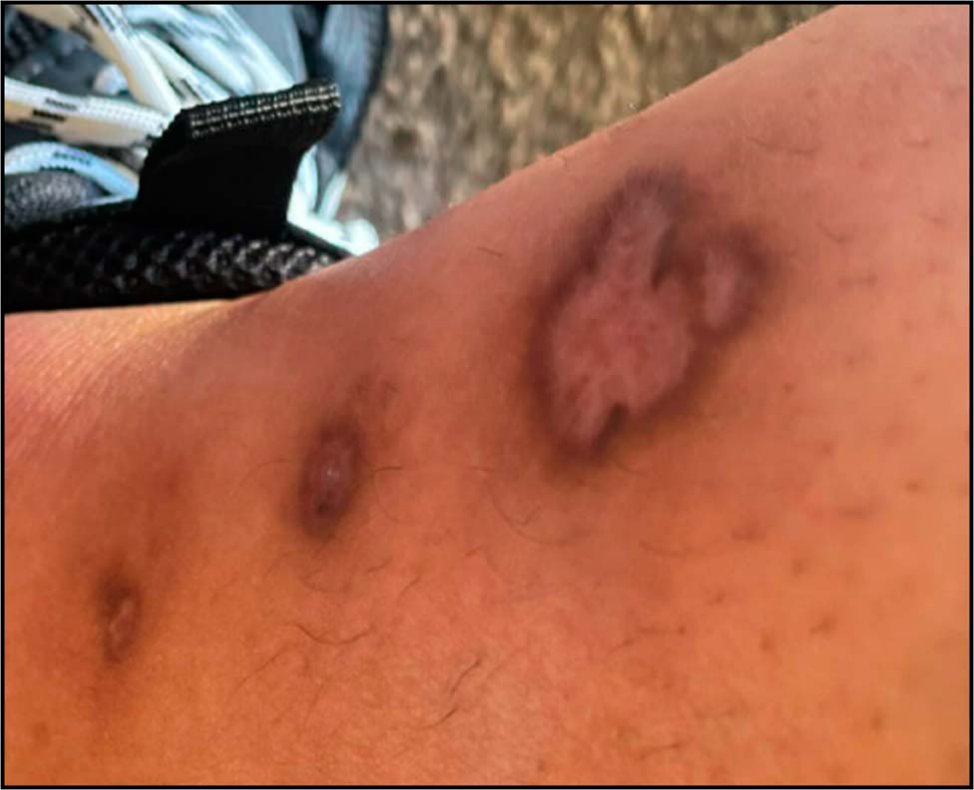
Medial view of the left lower extremity showing healing intercourse of classic ulcerative pyoderma gangrenosum.

**Figure 4.**
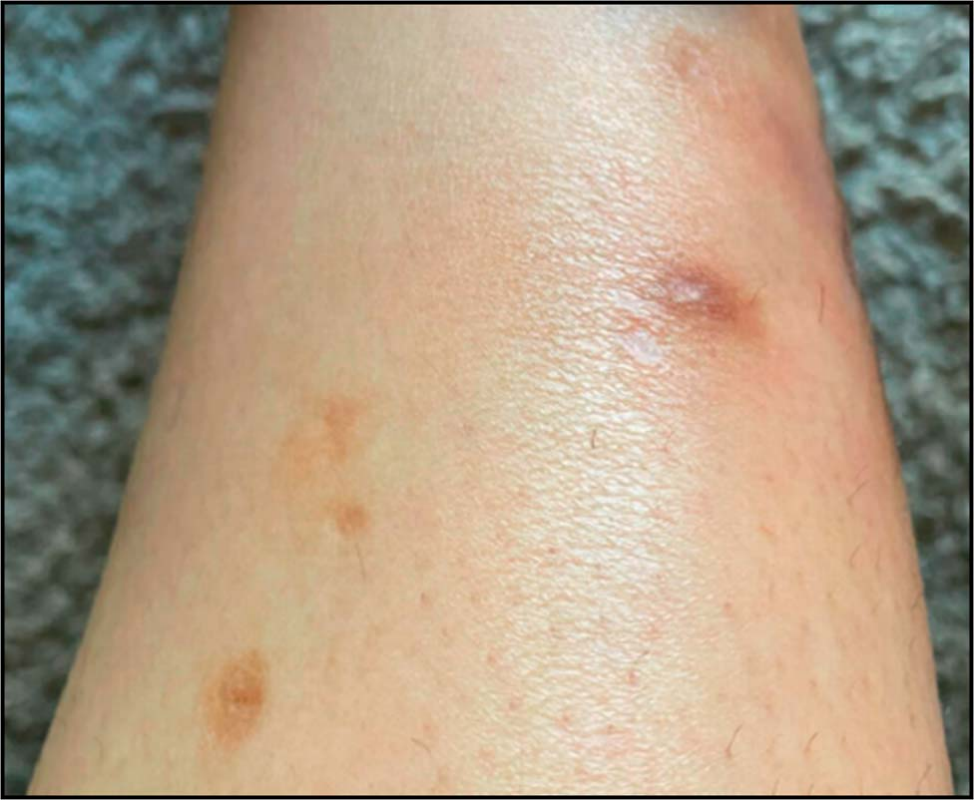
Lateral view of the left lower extremity showing healing intercourse of classic ulcerative pyoderma gangrenosum.

In the emergency department, she was diagnosed with cellulitis of the lower extremities and discharged on cephalexin, mupirocin, and trimethoprim/sulfamethoxazole. Pouchoscopy showed scattered ulcers in the J-pouch with normal small intestinal mucosa proximal to the J-pouch and moderately active histologic inflammation in the pouch. She was subsequently started on upadacitinib 45 mg daily for 8 weeks for presumed J-pouch inflammation and PG. At 7 weeks, PG exhibited significant healing (Figures 3 and 4). After 6 months of starting upadacitinib, her pyoderma gangrenosum has resolved without recurrence, and her pouchitis significantly improved.

## DISCUSSION

Over the past 3 years, the treatment of IBD has seen significant evolution of pharmacological treatments. One of the newest US Food and Drug Administration-approved medications for moderate-to-severe CD and UC is upadacitinib, a selective JAK1 inhibitor. JAKs are intracellular enzymes that transmit cytokine or growth factor signals involved in a broad range of cellular processes, including inflammatory responses and immune surveillance. The JAK family of enzymes contains 4 members—JAK1, JAK2, JAK3, and TYK2—that work in pairs to phosphorylate and activate signal transducers and activators of transcription (STATs). This phosphorylation in turn modulates gene expression and cellular function. JAK1 is important in inflammatory cytokine signals.^2^

PG, a neutrophilic dermatosis affecting the skin, is an uncommon dermatologic extraintestinal manifestation of IBD.^3^ The lesion usually begins as a papule or pustule at a site of trauma with a surrounding violaceous and undermined border with subsequent necrosis of the dermis resulting in deep ulcers as the lesion progresses. The incidence of PG ranges from 0.4% to 2.6%, and it is more common in women, those with other extraintestinal manifestations and those with colon-predominant inflammation.^4,5^

Patients with IBD may present skin lesions that interfere with quality of life, including erythema nodosum and pyoderma gangrenosum, which may or may not be associated with IBD disease activity. PG can be confused in the initial stages with minor trauma, infection, or cellulitis; that along with a nonestablished treatment creates challenges in its management. Our case highlights a delay in appropriate treatment due to concern for infection.

Upadacitinib has shown effectiveness in resolving EIMs in patients with UC after induction treatment with 45 mg and after maintenance with either 15 or 30 mg, with the 30 mg dose providing statistically significant improvements.^6^ Our case describes a patient with acute on chronic pouchitis at the time of PG diagnosis. Her overall gastrointestinal symptoms and skin lesions were significantly improved after 7 weeks of upadacitinib treatment.

This case shows the novel use of a therapy to treat both underlying IBD and PG. We describe the successful treatment of pyoderma gangrenosum with pouch inflammation with upadacitinib.

## DISCLOSURES

Paula Milena Prieto Jimenez is the article guarantor.

Author contributions: PM Prieto Jimenez: Manuscript draft, design and literature review. S. Tabib and B. Abbott: Manuscript review. G. Melmed: Manuscript draft and review.

Financial disclosure: None to report.

Informed consent was obtained for this case report.
